# From Lexicon to Flexicon: The Principles of Morphological Transcendence and Lexical Superstates in the Characterization of Words in the Mind

**DOI:** 10.3389/frai.2021.788430

**Published:** 2022-02-23

**Authors:** Gary Libben

**Affiliations:** Department of Applied Linguistics, Brock University, St. Catharines, ON, Canada

**Keywords:** mental lexicon, psycholinguistics, morphology, compounds, morphological transcendence, morphological superstates

## Abstract

The field of mental lexicon research has benefitted greatly from the founding metaphor of a dictionary in the mind. That metaphor, however, had its origins in a perspective in which the lexicon was seen as a static repository of representations with fixed structural properties. This paper presents a contrasting view. It is a view that highlights that words are activities that we perform, rather than simply representations that we have. It is proposed that lexical representations are best seen as hierarchies of action within a highly interconnected and dynamic system. The paper presents two principles of lexical organization: *morphological transcendence* and *lexical superstates*. The former principle claims that through the activities of language comprehension and production, lexical forms can develop variant forms. Thus, the form *key* may develop into forms such as *key*- (e.g., *keyboard)* and *-key*, (e.g., *turnkey*). The paper also discusses how transcendence leads to lexical superstates, which do not have a fixed morphological structure. As part of a lexical superstate, alternative morphological structures exist as potential realizations. Which one is actually realized will depend on the specific circumstances of a lexical action. An account is presented in which the effects of semantic transparency are treated in terms of transcendence and superstate interactions. It is claimed that this approach, which highlights the dynamic and flexible nature of the mental lexicon, has implications for how we approach the modeling of language and cognition in general.

## Introduction and Overview

In the history of psycholinguistics, the *mental lexicon* has been an extremely successful and enduring construct. One reason for its success is that it was built on a metaphor that is both familiar and easily accessible to all—a dictionary (Aitchison, 2012). Historically, the metaphorical relationship between the mental lexicon and a physical dictionary has made it easy for theoretical and computational linguists, psychologists, and neuroscientists to engage in transdisciplinary research. One reason for this is that it has enabled researchers to employ a common research vocabulary based on a shared metaphor. That research vocabulary could include terms such as *lexical store, lexical entry*, and *lexical access*. Another reason for the success of the dictionary metaphor is that it seemed to lead researchers to the kinds of activities that would need to be understood. For example, building on the observation that a physical dictionary enables users to access meaning through entries that are indexed by their form characteristics, it was easy to consider the mental lexicon to be a gateway to semantics. It was also easy to consider it to provide translations across modalities because, like a physical dictionary, it could be assumed to enable access to the oral modality from the visual modality and vice versa. Reasoning of this sort has very likely been instrumental in enhancing the effectiveness of programs of research that have sought to address issues such as (a) the nature of information contained in a lexical representation, (b) how lexical knowledge is conditioned by experience, (c) the means by which lexical representations are linked to one another, and (d) the extent to which characteristics of the mental lexicon vary across the lifespan and across populations.

From the outset, questions (a) through (d) have framed key research goals and provided important markers of progress. Since the term *mental lexicon* was first used by Treisman (1961), a great deal of progress has been made in addressing these questions. However, a great deal more needs to be understood in order to answer them. The goal of this paper is to advance that understanding by showing how the interaction of two key principles of lexical representation can capture dynamic properties of the mental lexicon. The two principles are *Morphological Transcendence* and *Lexical Superstates*. I present the case that, together, these two principles make it possible to understand how theoretical characterizations of the morphological structure of words are related to cognitive representations and cognitive activity in lexical processing. I also claim that the interaction of these principles can account for the effects of semantic transparency within words and associations among them. A key feature of this approach is that it highlights the manner in which a cognitive account of lexical knowledge must have, as its core, a focus on words as actions, rather than words as static representations. This highlights the way in which words are themselves processes rather than simply the objects of processing. It also highlights the dynamic nature of the lexical system and how a language user's experience can affect word knowledge and lexical interconnectivity.

As has been noted above, mental lexicon research has benefited greatly over its history from using a physical dictionary as a metaphor. It seems very plausible to expect that, at present, benefits can also be achieved in the opposite direction. Thus, dictionary creation can make use of metaphors that are grounded in cognitive activity and which foreground, in particular, how human lexical representations are dynamic and often structurally indeterminate.

## Words and Hierarchies of Action

In everyday speech, the term *word* is used with an ease that might lead one to think that it is completely unproblematic. This ease of use can be seen in idiomatic expressions such as *don't mince words, eat your words'*, and *famous last words*. In all three examples, the meaning of *words* seems both obvious and clear. Yet, an exact definition of *word* that holds across languages and across modalities of language use has proven to be quite elusive. In an approach that contrasted with the morpheme-based approach of Halle (1973), Aronoff (1976) argued that words constitute the fundamental units of representation and word formation. The matter of how to define the notion of word within linguistic theory was taken up by William (1981) and Di Sciullo and Williams (1987). Many contributions since that time have noted that the notion of word may not be as univocal as it first appeared (e.g., Haspelmath, 2011; Wray, 2015; Plag, 2018). This challenge of definition has been addressed by Mansfield (2021) through the adoption of an information-theoretic approach to wordhood which highlights the manner in which words are associated with higher levels of internal predictability. This gradient approach, which has its origins in Shannon's (1948) concept of entropy, offers a method that, by its nature, can be applied across a range of languages and lexical structures.

Within the context of mental lexicon research, the challenge of characterizing wordhood has traditionally been dealt with by conceiving of words in the mind as those lexical structures that are committed to memory (e.g., Aitchison, 2012). These may correspond to both monomorphemic and multimorphemic lexical structures and perhaps also multi-word sequences (Tremblay et al., 2011; Jeong and Jiang, 2019). Crucially, the construct of word within mental lexicon research must make reference to the kinds of cognitive activities that words both constitute and participate in. It is this perspective that is at the core of my claim that words are best seen as hierarchies of action. I assume that words are represented in the mental lexicon as a consequence of repeated cognitive activity. Thus, there is an important way in which words are best conceived of as those activities.

It is assumed that the exact nature of lexical action can vary in accordance with the morphological properties of individual languages. The consequence of this assumption is that the types of lexical structures in an individual's mental lexicon will be determined by the particular set of languages spoken. Thus, an individual's development of bilingualism may be accompanied by an expansion of the types of lexical organizations that can constitute a word for that individual. In all cases, however, those organizations are best seen as hierarchies of action. This is a term that highlights two additional and important properties of lexical actions—namely that they often exhibit complex internal structure and that they function as addressable elements within the cognitive system that we refer to as the mental lexicon.

## The Development of the Mental Lexicon as a Psycholinguistic Construct

The construct of a mental lexicon as a dictionary in the mind can be seen as part of the early days of neurolinguistics in the nineteenth century. The theorizing of Wernicke (1874) and the development of an explicit information flow model by Lichtheim (1885) contained the view that distinct centers in the brain can be seen as containing representations of lexical information. Because these approaches sought to account for specific patterns of aphasia, the modality specificity of lexical knowledge was a key consideration. Similar reasoning can be seen in twentieth century approaches to the modeling of the mental lexicon by reading researchers who sought to provide an account of differing patterns of acquired dyslexia. In that context, the logogen model of Morton (1969) was extremely influential. The logogen model represented the lexicon in a flow chart manner such that separate representations could be posited for lexical input and output in specific modalities. The logogen, the basic unit of the model, was assumed to be an evidence collecting element that had an activation threshold that responded dynamically to experience. In this way, the logogen model was able to capture long term effects of lexical frequency in which it is observed that words that are more common in the language are recognized more quickly. Later versions of the model (Morton, 1979) were designed to also capture modality-specific short-term repetition effects such as those seen in repetition priming experiments. The logogen model played a key role in the development of models of deep and surface dyslexia (e.g., Marshall and Newcombe, 1973; Morton and Patterson, 1980) and models that sought to link these to visual lexical processing, more generally (e.g., Coltheart, 1981). It was also foundational to the development of the computationally implemented Dual-Route Cascaded Model of Coltheart et al. (2001).

A second stream in the development of conceptions of the mental lexicon has its origins in the theoretical linguistic literature and in later concentrations on morphology as a sub-discipline. Theoretical approaches were, unsurprisingly, somewhat less concerned with modality-specific characteristics of lexical knowledge and the need to distinguish between input and output representations. Bloomfieldian linguistics already contained a notion of a lexicon, which was essentially an appendix to the grammar (Bloomfield, 1933). This approach to the role of the lexicon in the overall language system was also evident in Chomsky (1957), who highlighted the way in which the construct of a lexicon was needed to capture irregularities in the language (on the assumption that those aspects of the language that were rule-governed could be incorporated into the grammar). Thus, in Chomsky (1957), the lexicon was assumed to contain simple monomorphemic words such as *jump*, but to not include inflected forms such as *jumps* and *jumping*, derived forms such as *jumper*, or compound forms such as *jumpsuit*. In the work of Chomsky (1970) and Halle (1973), the role of the lexicon in generative grammar became an object of theoretical development, with an assumption that the characterization of a language would need to include lexical rules and constraints on the forms that those rules could take. A good deal of attention in subsequent work (e.g., Aronoff, 1976; Selkirk, 1982; Di Sciullo and Williams, 1987) was devoted to determining the extent to which the lexicon could and should be considered to be an autonomous component of the overall grammatical system, the extent to which lexical forms are idiosyncratic, and the extent to which lexical patterns are distinct from syntactic patterns.

A third stream of evidence in the development of the construct of a mental lexicon came from studies that used chronometric approaches to psycholinguistic experimentation and focused on issues of lexical access. This stream included studies of whether the mental lexicon contained separate representations for inflected forms of a word (e.g., Bertram et al., 2000; Raveh and Rueckl, 2000), whether derived and compound words were accessed in terms of their constituents or as whole forms (McQueen and Cutler, 1998), and if they were, whether that constituent access would occur before whole word access (pre-lexical decomposition) or after it (post-lexical decomposition) (e.g., Libben et al., 2003). There was also a considerable amount of research that focused on the nature of relations within the mental lexicon and pan-lexicon effects. For example, studies such as those reported in Forster (2004), Holcomb and Anderson (1993), and Neely et al. (1989) sought to determine how words that were related to each other semantically were represented in the mental lexicon. Other studies focused on factors such as orthographic and phonological neighborhood density (e.g., Ferrand and Grainger, 1992; Ziegler et al., 2003; Westbury and Hollis, 2007).

## The Field of Mental Lexicon Research Has Reached a Critical Juncture

The recent history of psycholinguistic research on the mental lexicon was approached by Kuperman et al. (2021) using structural topic modeling. They examined word usage during the 30-year period of 1990 to 2020 by including, as input to the modeling process, all abstracts of the 1,104 papers that were listed in the Web of Science database and which contained the keyword “mental lexicon” since 1990. They also included all 199 articles published in *The Mental Lexicon* journal since its creation in 2006.

The reasoning behind this approach is that topic modeling can provide a snapshot of a field, using the distribution of words used. In addition, it can also provide information related to the trajectory of a field (e.g., Hall et al., 2008; Cohen-Priva and Austerweil, 2015). Within such a perspective, the history of a scientific field can be seen as a type of time series analysis, in which future events in time are conditioned by prior events. In this way, the topic modeling approach to the mental lexicon reported in Kuperman et al. (2021) accords with the historical approach taken above. Fields of scientific research must grow and change. But the domains in which they develop are influenced by the foundations of the field and the trajectories that have emerged from those foundations. At key junctures, a field may engage in self-assessments that result in new directions. The field of mental lexicon research appears to be at such a juncture.

Structural topic modeling (Rosen-Zvi et al., 2004; Griffiths et al., 2007; Blei, 2012) enables the user to create topic labels that correspond to lexical clustering patterns within texts. Thus, as Kuperman et al. (2021) note, such labels can only be seen as short-cuts. Thus, it should not be assumed that the analysis covers the full range of the topic label. The topics that Kuperman et al. (2021) used in their analysis are shown in Table 1. Within each category of the table, they report the top five words that correspond to each of the three metrics.

**Table 1:** The Mental Lexicon topics represented as collections of most diagnostic words as reported in Kuperman et al. (2021).

**Topic**	**Category 1:** **Words with the highest probability in topic**	**Category 2:** **Words used frequently and exclusively in topic**	**Category 3:** **Words used frequently in topic, given their distribution**
*1. Theory*	mental, lexicon, model, research, cognit	express, health, social, psycholog, approach	belief, feel, depress, health, care
*2. Masked priming*	prime, morpholog, word, experi, effect	prime, root, mask, morpholog, hebrew	prefix, prime-target, mask, prime, soa
*3. Bilingualism*	languag, speaker, english, bilingu, nativ	learner, bilingu, nativ, colloc, speaker	efl, learner, foreign, colloc, bilingu
*4. Word recognition*	word, frequenc, effect, lexic, recognit	frequenc, neighborhood, competit, respons, recogni	deaf, neighborhood, densiti, neighbor, estim
*5. Neuroscience*	process, lexic, activ, semant, brain	left, patient, tempor, brain, neural	lobe, gestur, magnet, gyrus, cortex
*6. Children*	children, group, read, age, studi	children, age, abil, skill, score	peer, dyslexia, year-old, dyslex, month
*7. Speech*	speech, phonolog, word, represent, model	speech, phonet, syllabl, sound, phonem	tone, tonal, acoust, voic, syllabl
*8. Inflection*	form, verb, inflect, regular, morpholog	irregular, regular, verb, inflect, plural	irregular, participl, plural, tens, singular
*9. Chinese/Japanese*	word, compound, read, mean, chines	compound, chines, sens, constitu, charact	colleg, compound, polysem, self-pac, chines
*10. Syntax*	noun, semant, categori, name, context	gender, grammat, phrase, idiom, categori	bare, idiom, gender, block, phrase

*The order of categories (1–10) is arbitrary.*

*Source: Adapted from Kuperman et al. (2021) (https://benjamins.com/catalog/z.238)*

**Figure 1:**
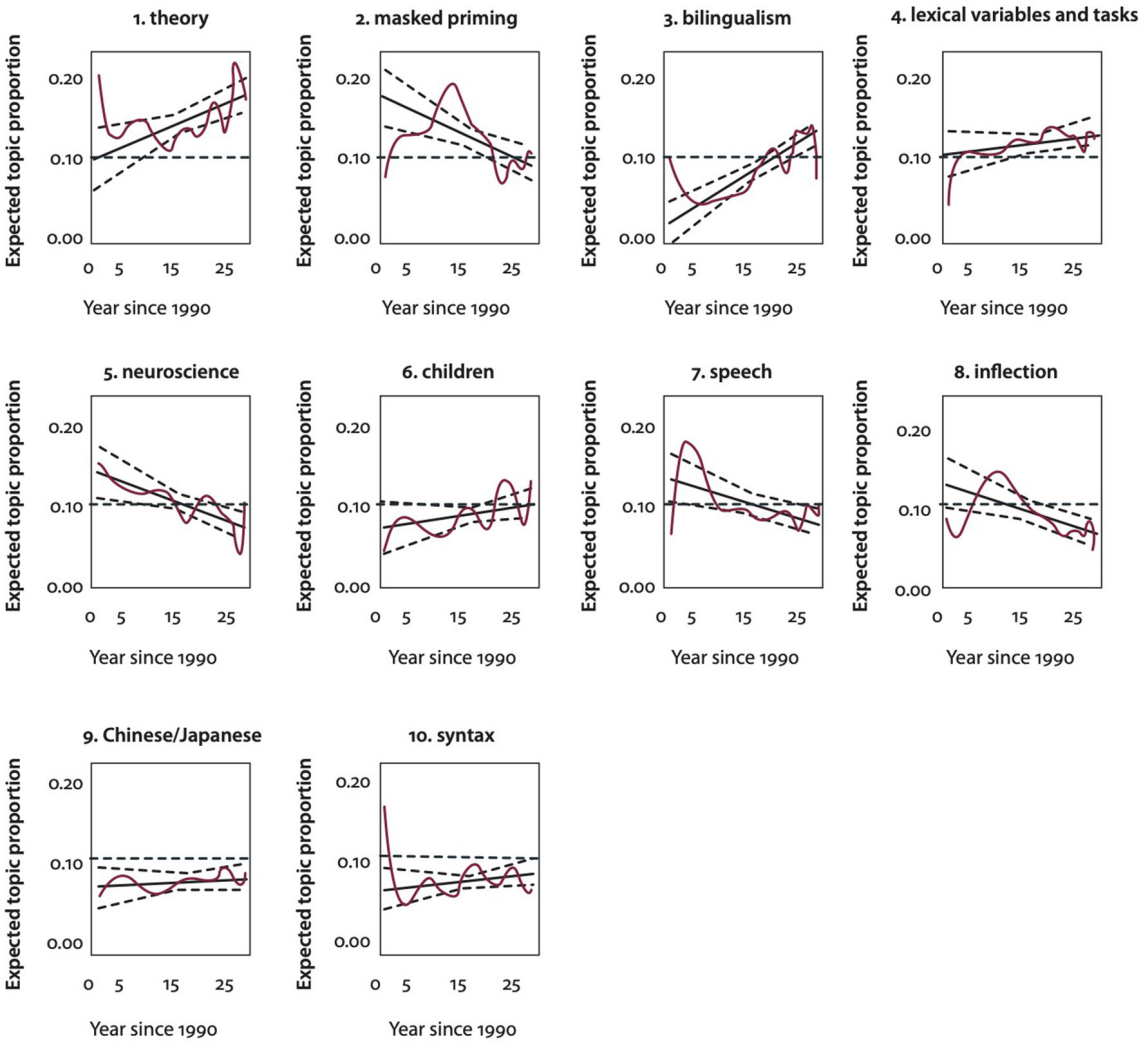
Change in topic prevalence since 1990 reported in Kuperman et al. (2021). The non-linear fit is shown in red, the linear fit in black (dotted lines mark the 95% confidence interval). The horizontal (green) dotted line shows the default 10% of the topic. That would be expected over 10 topics. Source: From Kuperman et al. (2021) (https://benjamins.com/catalog/z.238).

In Figure 1, the trajectories of each of the 10 topics are displayed. As can be seen in this figure, three of the 10 topics are above the 10% level, which represents the default expectation for 10 topics over the corpus as a whole. Those three topics are *theory, bilingualism*, and *lexical variables and methods*. Of these three, the topic of *theory* stands out as dominant. As can be seen in the figure, the topic has experienced a steep rise over the past 20 years. The perspective taken in this paper is that the very substantial rise in the relative prominence of themes that can be grouped under the topic of *Theory* is consistent with the view that the field of mental lexicon research is at a critical juncture. That critical juncture is one in which the benefits that the field has received from the metaphor of the mental lexicon as a physical static dictionary and the conceptualization of words as fixed representations has enabled a sufficient accumulation of knowledge so that it is possible to advance the construct of a *mental lexicon* and the construct of a *lexical representation* in ways that are more dynamic, more sensitive to sources of individual variation, and therefore more psychologically aligned. As I claim in the following sections, key components of such an advancement involve considering words to be hierarchies of action within a highly interconnected mental lexicon. As is further detailed below, I claim that two principles of lexical organization, *morphological transcendence* and *lexical superstates*, are important in the understanding of the underlying nature of lexical knowledge.

## The Dynamic Nature of the Mental Lexicon and a Conceptualization of Words as Actions

The construct of a mental lexicon and the fundamental nature of lexical entries have received substantial scrutiny and challenge over the past decade. Elman (2011) in a provocatively titled article, *Lexical knowledge without a lexicon?*, challenged the extent to which it is advantageous to posit a separate lexical component of the language system. The challenge of Baayen et al. (e.g., Baayen et al., 2011, 2019) has focused on the need to posit morphemes as units of representation in the mental lexicon and the alternative advantages of modeling the lexicon and lexical processing using naïve discriminative learning.

Libben (2017, 2019) has claimed that the static nature of the metaphor that has been used for the constructs of a mental lexicon and for lexical entries within it have hindered the ability to model the manner in which the mental lexicon of an individual can change over time (particularly in the case of bilingualism) and the ways in which the nature of a lexical entry can change in response to specific processing needs. Lexical knowledge increases dramatically in childhood and continues to grow throughout the lifespan. The acquisition of one or more additional languages can greatly expand the number of words known as well as the patterns that correspond to lexical structures.

Another key difficulty associated with treating the mental lexicon as though it were a static dictionary in the mind is that it leads to a problem often termed *the homunculus problem*. The term was coined by Kenny (1971) and has since been at the center of considerable discussion in the philosophy of mind and neuroscience (e.g., Dennett, 1992; O'Reilly et al., 1999; Rosas, 2014; Rowe, 2021). The essence of the homunculus problem is this: If an explanation for how the brain interprets something requires an interpreting agent within the brain, then the phenomenon has not really been explained. Rather, the explanation has simply been delayed. In this way, the postulation of a mental lexicon as a type of dictionary in the mind can create a homunculus problem because it seems to require that there be an interpreting agent (i.e., a little scholar) in the brain that would be consulting that lexicon.

The homunculus problem in relation to the mental lexicon can be seen as a specific instance of a more general problem of positing mental representations that need to be consulted. For example, it is difficult to explain reading by positing representations of letters in the brain that need to be read and it is difficult to explain speech production by positing phonemic representations that need to be translated into motor movement. Thus, it may be that the problem to overcome in modeling mental representations in the mental lexicon is simply the assumption that they are representations.

As has been noted above, there is a strong tendency in the consideration of language processing to single out words as objects that are drawn upon in the acts of language comprehension and production. This tendency can be seen in the aphasiological literature beginning with Wernicke (1874), the linguistic literature beginning with Bloomfield (1933) and through much of the psycholinguistic literature on lexical processing (Libben, 2017). Upon deeper consideration, however, it may turn out to be the case that term *word* is simply the name that we give to hierarchies of lexical action. Under such a view, words are things that we do. A word label (or lexical entry) can be considered to be a shorthand term for a hierarchy of action, in much the same way that the term *U-turn* is a shorthand for a particular hierarchy of driving actions or the term *pass* is a shorthand term for another hierarchy of driving action or a shorthand term for hierarchies of action displayed among football or ice hockey teammates. This perspective allows us to consider words as patterns of lexical action and the mental lexicon as a system of lexical action. The lexical actions that correspond to the everyday notion of *word* are the actions that we perform when understanding a word that is seen or heard and when using a word in oral or written production. Our ability to draw upon these practiced actions as though they were discrete things enables fluency of language use and supports the interconnectedness of lexical actions within the mental lexicon.

Conceptualizing words as hierarchies of action and conceptualizing the mental lexicon as a system of lexical activity leads to a view that is quite distinct from that which could be associated with a traditional, bound, desktop dictionary. It is not, however, incompatible with the kinds of activities that are currently possible in the digital realm of dictionary creation.

This brings us to the question of what is to be gained by re-metaphorizing the mental lexicon as a system of lexical action and re-metaphorizing words as hierarchies of action. In my view, the general answer to this question is *flexibility*. Language users gain new words throughout their lives. There is considerable evidence that the dominant characteristic of the mental lexicon is its massive interconnectivity. It is a system in which, in principle, every element could be connectable to every other element. It has been estimated that a native speaker of English has a vocabulary of 50,000 words (Brysbaert et al., 2016). In such a system, the addition of a single word to the system could create up to 50,000 new connections. Even if the new word were connected to only 1% of existing words, that would result in 500 new connections (see Steyvers and Tenenbaum, 2005, for an analysis of patterns of growth within semantic networks).

Within the dynamics of such a system, the identities of the elements themselves characterize the system much less than does the nature of the connections that are possible among them. Such a system would always be active and in flux as new elements are added and, most importantly, as new connections are developed Thus, it may be the case that *we can never step into the same mental lexicon twice*.

## Lexical Action Creates Morphological Transcendence: Compound Words as the Test Case

Thinking about words as actions can have a substantial impact on how we approach the mental lexicon, lexical access, and the modeling of lexical processing. If indeed, a lexical representation is essentially a shorthand term for a hierarchy of lexical actions, and if these actions change in accordance with specific processing demands and contexts, it follows that a lexicon created by lexical activity will have considerable partial redundancy and a proliferation of elements.

Libben (2014) has claimed that such a process can be seen particularly clearly for compound words. These are words that are themselves composed of words that are likely to be free-standing in the language. The need to account for how words can exist within words extends back to Aristotle, who considered compound words to be problematic for his assertion that words are atomic units. Aristotle's solution was to claim that compound constituents are not actually free morphemes, but rather become altered by virtue of their existence within compounds. Essentially, this is also the approach taken by Libben (2014) in the postulation of *morphological transcendence*.

Compound words may offer the ideal test case for transcendence and, more generally, an ideal window to the functional architecture of the human lexical system. Compound words are both common and productive across the world's languages (Dressler, 2006). It is quite possible that they are the core human word formation mechanism—the one that has, from the outset, enabled languages to develop new words from existing words. The fact that compounding most typically involves making words from other words enables us to consider morphological operations and morphological constituents, while avoiding some of the complications associated with the question of whether morphemes are useful theoretical and processing constructs (see Baayen et al., 2011, 2019; Blevins, 2016). This consideration is critical in that it means that the morphological structure of a compound word must begin as an association among words. It is only a highly interconnected mental lexicon that can support this. Moreover, because compounding is typically very productive, the probability that a language user will encounter an unfamiliar compound word during everyday language processing is high. That unfamiliar compound word can only be understood in terms of its constituents (which, unlike affixes, are not members of a small restricted set). Thus, it is also the case that compound processing requires that morphological interpretations be created through lexical links accessed in real time.

The term *transcendence* as used by Libben (2014) refers to a process in which a form, which exists as a free-standing word can also become a compound constituent as a result of being used in the production and comprehension of compound words. When this happens, it can be said that a new lexical entity is being created because the lexical system new has a form that is associated with a specific role within compound words. Consider, for example the word board as a free-standing word. As is not at all unusual for English words, the word *board* is polysemous. So, let us assume that we are referring to the meaning of board as a flat surface of wood. Even with that specificity, it seems clear that the meanings of-*board* in the compound words *surfboard, snowboard*, and *wakeboard* have deviated somewhat from the meaning of board as a free-standing word. At the same time, they constitute a family in which the three instances of-*board* in these compounds are very close in meaning and share the property of being something that is ridden recreationally. In this way, we can say that *-board* is a morphological entity created through the lexical activity of compound processing. A similar pattern can be seen in the case of the compounds *pressboard, chipboard*, and *particleboard*. Here too, the compound constituent *-board* is clearly linked to the free-standing word *board* and the meanings “flat surface” and “wood.” Nevertheless, we see again that the compound constituent meanings differ from the whole word meaning much more than they differ from each other. In this way, their final constituent, *-board*, can be said to be transcended.

It is important to note also that the compound constituents that correspond to *board* in the compounds *snowboard*, and *paddleboard* and the compound constituents that correspond to *board* in the compounds *boardroom* and *board table* have position-specific properties. Thus, the principle of morphological transcendence results in a proliferation of lexical elements such that the single free-standing word *board* becomes three lexical elements *board, board-*, and *-board* (where the hyphen is used to signify the position of the constituent within a compound). Of course, this proliferation is further increased when one considers other meaning families of *board* and their roles within compound words (e.g., *whiteboard and blackboard; chessboard and checkerboard; leaderboard*, etc.).

Günther and Marelli (2021) present an implemented computational system to represent role-dependent constituent phenomena and to test the predictions of morphological transcendence. The behavior of the system supports many of the predictions of transcendence and also reveals new system-based phenomena. Moreover, the Günther and Marelli (2021) approach accords with a key underlying assumption of morphological transcendence, namely that the propensity for the proliferation of lexical elements is evidence that the lexical system is not organized and does not function in order to maximize efficiency. Rather, it seems to be designed to maximize opportunity for lexical activation and lexical activity. This a perspective introduced by Libben (2006) as the *Principle of the Maximization of Opportunity* to capture how the human language processing system has developed to enable the creation of as much meaning as possible.

**Figure 2:**
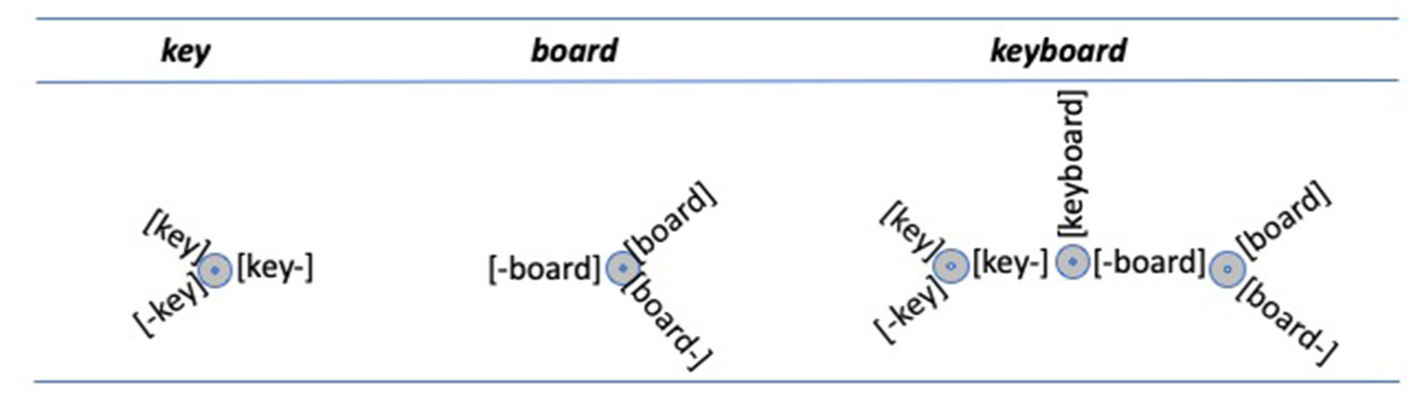
Morphological transcendence creates a proliferation of lexical entities. The representations of key, board, and their combination in the compound keyboard.

Figure 2 displays an example of how morphological transcendence results in the proliferation of lexical entities. As can be seen in the figure, the words *key* and *board* each have transcended position-specific variants. It is claimed that these variants are the result of a person's experience with compounds and compound families. So, for a university instructor, it might be expected that the family of *whiteboard, chalkboard*, and *noticeboard* will be dominant over the family of *particleboard, pressboard*, and *chipboard*. For a building contractor, the reverse relationship might exist.

The representations in Figure 2 display possibilities for individual persons, rather than structures for individual words. It is assumed that the position-specific transcended elements develop over time as a particular morphological family grows and/or as an individual gains greater experience with the family. Thus, for example, a person could encounter the word *surfboard* and shortly thereafter build a family that includes *wakeboard, snowboard*, and the potential compounds *riverboard* and *aeroboard*. In this way, the strength of the transcended constituent *-board* is related to the number of compounds in which it is a final constituent and the frequency with which those compounds are used.

The effect of morphological transcendence is often particularly salient when a novel compound is created that contains a transcended constituent with a strong positional family. The initial constituent *bat-* is an example of this. As a result of the creation of the comic book character “Batman” in the late 1930s and the appearance of a television series, movies, and graphic novels, a great many compounds beginning with *bat-* have been created (of which *batmobile* and *batcave* are among the most frequent). The consequence of this can be seen by conducting a Google image search for the compound *batboard*. The resulting search will likely yield almost exclusively Batman-related objects and likely not any images related to a bat as a flying mammal. Interestingly, these images will span difference senses of the constituent -*board*. Thus, *batboard* can be seen to refer to Batman-themed surfboards, skateboards, electronic skateboards, computer keyboards, and musical keyboards. It is the process of transcendence that makes this kind of lexical creativity and morphological productivity possible. Transcendence also makes it possible for the phenomenon to be accounted for within psycholinguistic models of lexical processing.

## Morphological Parsing Without a Parser

It has been claimed above that lexical action creates morphological transcendence, which results in the proliferation of transcended compound constituents. That lexical action can involve a variety of operations. Lexical entities must be accessed and new links may also need to be created. Particularly in the case of the comprehension of novel compounds, compounds would need to be parsed into potential constituents. This parsing process carries with it another inherent homunculus problem for, just as it is unlikely that there is a little scholar in the mind consulting the mental lexicon, it is unlikely that there is a little morphologist who is performing (or reading) morphological parses. One could, of course, resort to the assumption that embedded words just emerge through activation, but it seems unlikely that an unconstrained process of this sort would yield psychologically realistic effects. If it did, then we should expect the processing of a novel compound such as *riverboard* to result in the activation of substrings such *verb, boa, boar*, and *oar*.

**Figure 3:**
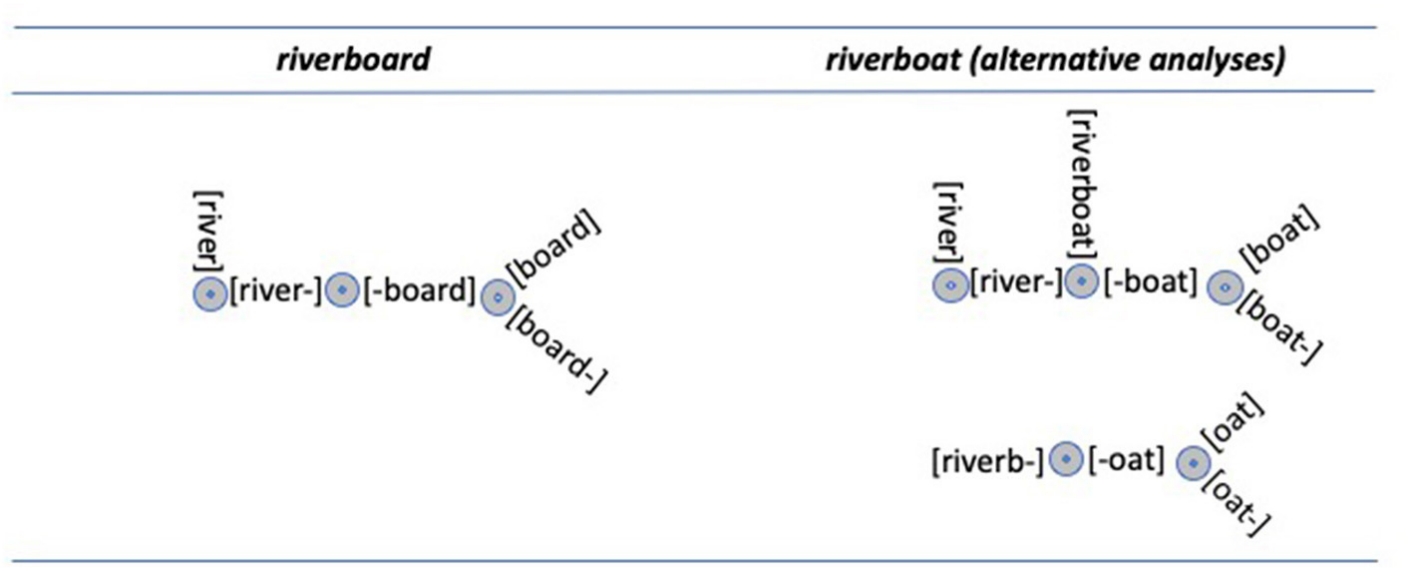
The compounds riverboard and riverboat, showing results of lexical splitting which is triggered by a left or right substring being an existing word in the mental lexicon. It is assumed that that there is no transcended constituent that could be represented as [–river].

It is assumed here that the psycholinguistic morphological structuring of compounds is achieved through a simple splitting operation that has substantial consequences for the lexical system as a whole. That splitting operation simply enables a word to have two components. It is driven by an initial or final substring “popping out” so that the entirety of the string is used. As a result, in the case of *riverboard*, the substrings *river* and *board*, would be activated, but the substrings *verb, boa, boar*, and *oar* would not be activated. It is important to note that we expect that novel compound recognition does not require that both the putative initial and final constituents be words. If that were the case, an unfamiliar compound such as *boysenberry* would not be understandable as a type of *berry*. It is furthermore assumed that all possible (*n* – 1) splits can be carried out, so that the processing of a compound such as *riverboat* could potentially result in the activation of *river, boat*, and *oat*. Figure 3 shows these configurations for the compounds *riverboard* and *riverboat*.

## Why Do We Understand the Word *Flexicon*?

The discussion of morphological parsing above simply proposes that language users split a word in two when either a left or right substring corresponds to an existing word in the individual's lexicon. It is possible that such a simple operation could stand behind morphological processing in general and explain the propensity for morphological structures to be arranged in binary hierarchies. This simple operation may also stand behind our ability to produce and understand words such as the neologism *flexicon*, used in the title of this paper. Despite the fact that the word does not seem to have a well-formed morphological structure, both the substrings *flex* and *lexicon* can be activated when reading it. This result falls out from the assumption that constituent splitting is triggered by the existence of a word in the mental lexicon that corresponds to an initial or final substring. Thus, *flexicon* will be processed as the alternatives [flex][icon] and [f][lexicon], resulting in the activation of both the words *flex* and *lexicon*.

**Figure 4:**
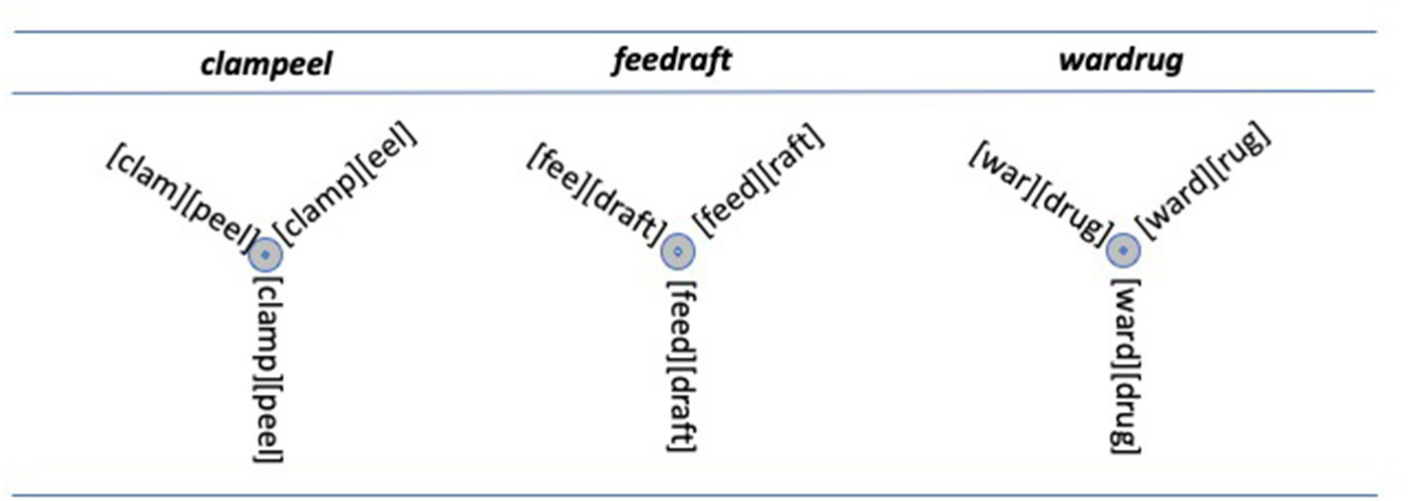
Ambiguous novel compounds. In each case, there are four possible lexical activations. The representations at the bottom indicate that participants are not constrained to produce well-formed parses but, rather, can create an interpretation from, for example, both clamp and peel (even though there is only one letter “p” in the input string).

Libben (2020) reported on the processing of a class of novel compound words that are particularly revealing of the effects of this type of morphological processing. The class of compounds can be referred to as ambiguous novel compounds. These are compound words that have been created by combining two words such as *clamp* and *peel*, for which the last letter of the first word and the first letter of the second word are the same (in this case, “p”). Additionally, the first word minus its last letter corresponds to an existing word of English and the second word minus its first letter corresponds to an existing word of English. In this way, ambiguous stimuli such as *clampeel, feedraft*, and *wardrug* can be created. In each case, four lexical constituents can be activated, as is shown in Figure 4.

Libben (2020) reports an experiment in which participants were presented with these words in a progressive demasking task and were required to type each word to indicate their recognition of it. Typically, in this task, compound words show elevated keystroke latencies at the constituent boundary, i.e., as the time taken to type the first letter of the second compound constituent (Libben et al., 2014; Gagné and Spalding, 2016). This was also what was observed by Libben (2020) for unambiguous novel compounds (e.g., *floataxe*). For the novel compounds, however, keystroke latencies were less elevated at the constituent boundary and, importantly, were equally elevated at both possible constituent boundaries (e.g., both before and after the “p” in *clampeel*).

This finding suggests an indeterminacy in the interpretation of words such as *clampeel*. I suggest that this indicates that there is a way in which the interpretation of *clampeel* has elements in common with the interpretation of *flexicon*. In both cases, all possible left and right constituents are activated, so that the opportunity of lexical activation is maximized.

This leads naturally to the question of which is the correct structure for *clampeel*: *clam-peel* or *clamp-eel*? In my view, the answer that best accords with the data and with what is known about the overall nature of the mental lexicon is that there is no actual structure, there are just structural possibilities. Indeed, the representations in Figures 2–4 are designed to capture those possibilities. In this way, rather than displaying lexical structures, they represent lexical superstates. These superstates are discussed in the following section.

## How can Lexical Superstates Change How we See Lexical Representations and the Mental Lexicon?

In the sections above, it has been claimed that our conceptualization of the mental lexicon and the nature of lexical representations can be advanced by considering words as actions and by foregrounding how the mental lexicon, as a dynamic knowledge system, changes as a result of individual experience in the production and comprehension of language. I have claimed that compound words offer a privileged window in this endeavor because they are words that themselves can be said to contain words. Moreover, almost any word of the language can become a compound constituent. Thus, compound morphology can serve as an instantiation of how everything can be connected to everything in the mental lexicon. In this way, the development of morphologically structured compounds falls out naturally from the principle of transcendence and the interconnectedness of words in the mind.

Throughout the discussion of compound representation and processing, lexical representations have been captured in configurations such as those shown in Figures 2–4. These figures do not show compound words or their constituents to have univocal structures, but rather represent them as structural possibilities, or lexical superstates (Libben, 2017, 2020).

The term superstate draws on a metaphor from quantum physics, which interestingly, itself draws on the psychology of William James (Hunt, 2001; Libben, 2017). One of the core features of James' perspective on thought and consciousness was his view that psychological constructs cannot be static entities (James, 1890). This is very much the perspective on the mental lexicon offered here.

The notion of lexical superstates enables us to frame issues of morphological representation of compound words in terms of two types of lexical action. The first type fosters activation across the lexicon and the proliferation of lexical representations. In the case of compound words, the presence of initial or final substrings that are known words triggers lexical activation of the substring and a splitting of the larger lexical structure that contains that substring. This process maximizes the opportunity for meaning creation and the development of multiple potential structures. The repetition of this process can create new transcended constituents that are position-specific and also role-specific within compound structures. The result of this is typically the activation of multiple representations that correspond to the compound and its constituents.

The second type of action involves the actual use of the compound as part of a language production or comprehension activity. Until this happens, we may consider the multiple representations to identify lexical possibilities. When one of them is actually used, however, there is the potential competition and conflict with the others. In this way, whole words that correspond to compound constituents and the constituents themselves can be interfering. This can be particularly evident in partial repetition priming experiments with compound targets because the prior presentation of a constituent as a whole word as the prime, then competes with its (often transcended) constituent representation (see Libben et al., 2018, for an instance of this with Hebrew and English compounds).

This perspective on lexical superstates may also provide a way to understand semantic transparency effects in the processing of compound words. There was early evidence that the recognition of compound words is sensitive to their semantic transparency (Sandra, 1990; Libben, 1998) and subsequent research has supported this conclusion (see Günther and Marelli, 2019; Sandra, 2020; Schäfer and Bell, 2020). The framework employed in the present paper may offer an account for why this effect obtains (i.e., why *gooseberry* is processed in a manner that is different from the way *blueberry* is processed). It may be the case that this falls out from the properties shown in Figures 2–4. In these figures, both the whole word representations of compound constituents (e.g., *blue* and *goose* for *blueberry* and *gooseberry*, respectively) and their transcended constituents (e.g., *blue-*and *goose-*) are part of the superstate representations of the compounds. Thus, when there are semantic differences (as would be the case for *goose* vs. *goose-* but not for *blue* vs. *blue-*), greater computation may result. In this way, the semantic transparency effects can be seen as deriving from a combination of the properties of morphological transcendence and lexical superstates.

## Implications: What Is a Lexicon? What Is a Word?

From the outset of this paper, it has been claimed that the character of the field of mental lexicon research has been shaped by its transdisciplinarity. In this way, it can be likened to the marketplace cities of the ancient world. These were places in which ideas, beliefs, customs, and cultures came into contact. They were places in which information flow was multidirectional and, thus, in which new understandings could develop.

There is good reason to believe that the field of mental lexicon research has also been a place in which new understandings could develop. That development has certainly been enabled and enriched by the culture of dictionary creation and the concepts and metaphors that could be shared from that culture. As has been noted above, at this point in the development of mental lexicon research, it is perhaps possible that some benefit could also flow in the opposite direction.

In my view, the title of this paper, *From lexicon to flexicon*, encapsulates important features of what has been learned and what could be applied from research on human lexical processing. We have seen that the mental lexicon is characterized by massive interconnectivity. We have seen that it is *psychocentric*, rather than *linguacentric*. In other words, mental lexicons belong to human minds, not to languages.

Perhaps the most important observation related to this point is the interconnectedness of the mental lexicons of bilingual persons, who make up the majority of the world's population (Grosjean, 2012). Recent research has highlighted the non-selectivity of bilingual processing. Words of one language prime words in another, translation equivalents cannot be easily suppressed, and bilinguals seems to be in possession of integrated lexicons (Kroll and Ma, 2017; Vaid and Meuter, 2017). The consequence of this is that people who speak more than one language are in possession of a single mental lexicon and that lexicon is not a lexicon of a single language.

I have claimed that the mental lexicon is best seen as a dynamic system of lexical activity. In such a system, there are no static things. Because the acquisition of new words continues through the lifespan (particularly for speakers of more than one language), and because all lexical representations within the lexicon can be related to all others, it follows that the lexicon is likely always changing. In this way, the mental lexicon may be considered to be the cognitive system within which lexical events take place and whose characteristics change as a result of those events.

The perspective advanced in this paper is that words are those lexical events. I have suggested that word representations can be seen as lexical superstates—a set of structural and interpretive possibilities for lexical events. When those events actually occur, though, alternative superstate possibilities can come into conflict. This perspective claims that our perceptions that words are static representations rather than actions may result, at least in part, from cultural conventions and technologies, including, most notably, the creation of the printed word.

The considerations above of the fundamental nature of the mental lexicon and lexical representation bring to the foreground that, even if words are events and lexical representations are hierarchies of action, it is difficult to refer to them in this way. It has thus been convenient to use representational descriptions as a type of shorthand for hierarchies of lexical action. As I have noted above, this has precedence in many domains. We refer to U*-turns* as though they were things, when in fact we know that they are actions. Similarly, a tennis instructor will talk to students about their *serve*, as though it were a thing, even though serves are actions.

Finally, it is important to note that the perspective presented here is one that goes beyond efficiency by positing the proliferation of lexical entities and connections among words. It is also a perspective that goes beyond determinacy by positing morphological superstates and claiming that multimorphemic words in the mind are morphologically organized, but that they do not have a definite morphological structure. In this way, their properties reflect those of the flexicon in general.

## Data Availability Statement

The original contributions presented in the study are included in the article/supplementary material, further inquiries can be directed to the corresponding author/s.

## Author Contributions

The author confirms being the sole contributor of this work and has approved it for publication.

## Funding

This research was supported by the Social Sciences and Humanities Research Council of Canada Partnership Grant 895-2016-1008 (Words in the World).

## Conflict of Interest

The author declares that the research was conducted in the absence of any commercial or financial relationships that could be construed as a potential conflict of interest.

## Publisher's Note

All claims expressed in this article are solely those of the authors and do not necessarily represent those of their affiliated organizations, or those of the publisher, the editors and the reviewers. Any product that may be evaluated in this article, or claim that may be made by its manufacturer, is not guaranteed or endorsed by the publisher.
